# Influence of Exposure Time and Driving Frequency on Cytotoxicity in In Vitro Ultrasound With Constant Mechanical Indices

**DOI:** 10.1002/elsc.70011

**Published:** 2025-05-15

**Authors:** Taigo Oyama, Chikahiro Imashiro, Yuta Kurashina, Keita Ando, Kenjiro Takemura

**Affiliations:** ^1^ School of Science for Open and Environmental Systems Graduate School of Science and Technology Keio University Yokohama Japan; ^2^ Department of Mechanical Engineering Keio University Yokohama Japan; ^3^ School of Engineering The University of Tokyo Tokyo Japan; ^4^ Division of Advanced Mechanical Systems Engineering Institute of Engineering Tokyo University of Agriculture and Technology Tokyo Japan

**Keywords:** cavitation, cell culture, sonochemistry, ultrasound

## Abstract

Sonochemistry has become increasingly important in bioengineering research, and many in vitro and in vivo bioapplications have been developed. Cytotoxicity is always a concern in its implementation. For in vivo treatments and studies, mechanical index (MI) is known to ensure biocompatibility, and even in vitro MI has been used. Because cell characteristics and acoustic phenomena differ in vitro and in vivo, we questioned using MI in vitro. The in vitro cytotoxicity of ultrasound exposure should be investigated to support the development of cutting‐edge sonochemistry. In this study, a system for irradiating cultured cells with 1–2 MHz‐range ultrasound was developed to demonstrate the invalidity of employing MI alone in vitro. The results showed that cell damage is defined by the MI, ultrasound frequency, and exposure time, which are new indices for quantifying cell damage. Furthermore, cavitation and acoustic streaming are shown to be the main scientific factors that injure cells.

## 1 Introduction

Interdisciplinary research has led to innovations worldwide, and acoustic engineering is also attractive in this context. Thus, many collaborations with other research fields, such as sonochemistry, have developed. Sonochemistry has attracted considerable attention in the bioengineering field. Ultrasound has been used for imaging, ultrasound surgery, and other in vivo treatments owing to its non‐invasiveness [1, 2, 3, 4]. Even in vitro ultrasound is gaining attention, with many applications using various intensities of ultrasound. Ultrasound microscopy and ultrasonic breaking have been employed in bioengineering workflows with extremely subtle and strong acoustic intensities, respectively [5]. Furthermore, according to recent studies, ultrasound with an intensity between that of ultrasound microscopy and breaking can be employed to develop cutting‐edge bio‐applications by controlling the physical positions or biochemical functions of cells. For position control, cells can be handled based on the acoustic tweezers theory [6, 7], and cell patterning, tissue generation, new culture methods, and lab‐on‐a‐chip technologies have been developed [8, 9, 10, 11, 12, 13]. Cell function, migration, angiogenesis, apoptosis, and differentiation can be regulated using mechanotransduction theory [14, 15, 16, 17, 18]. These bioapplications have been employed in broad medical studies such as cell therapy, regenerative therapy, drug delivery systems, diagnosis, and lab‐on‐a‐chip technology. In summary, sonochemistry is leading medical studies in bioengineering to rescue our lives.

**Summary**

• Without a doubt, the influence of ultrasound on cytotoxicity is crucial in all scenarios of bioengineering research field.

• The applications of ultrasound in bioengineering are diversifying.

• Even in vitro, ultrasound in life sciences and technologies gets attention, including cell culture passage, tissue engineering, transgenesis, and cell sorting techniques using ultrasound.

• As for cell culture passage, ultrasound has been utilized for enzyme‐free cell detachment, which results in an effective cell culture process.

• In the field of tissue engineering, acoustic tweezers techniques have been used to generate cell aggregation, and mechanotransduction studies have been conducted with ultrasound.

• One of the common methods for cell sorting is based on ultrasound trapping, and sonoporation has become a promising method for transgenesis.

• We, hence, believe our study is helpful for the development and study of bio‐applications.

Cytotoxicity has always been the center of attention in sonochemistry studies in bioengineering. For in vivo treatments and studies, the mechanical index (MI) (MI = *p /* $\sqrt{f}$, where *p* and *f* represent the acoustic pressure and the frequency, respectively) is known to ensure biocompatibility, and researchers and healthcare providers can follow the criteria of ultrasound intensity in their works [19]. MI has also been used in in vitro studies [20, 21], however, whether its usage in vitro is reasonable is yet to be determined. To support the development of cutting‐edge sonochemistry, the in vitro cytotoxicity under ultrasound exposure should be investigated independently. To break away from MI, we developed an experimental system evaluating the cytotoxicity under certain ultrasound conditions and demonstrating that cytotoxicity for in vitro cells can be determined by MI, ultrasound frequencies, and exposure time. Using MHz frequency ultrasound, we also identified the scientific factors that introduce cell death into cavitation and acoustic streaming in our experimental setup.

## 2 Results

### 2.1 Development of Ultrasound Irradiation System

**FIGURE 1:**
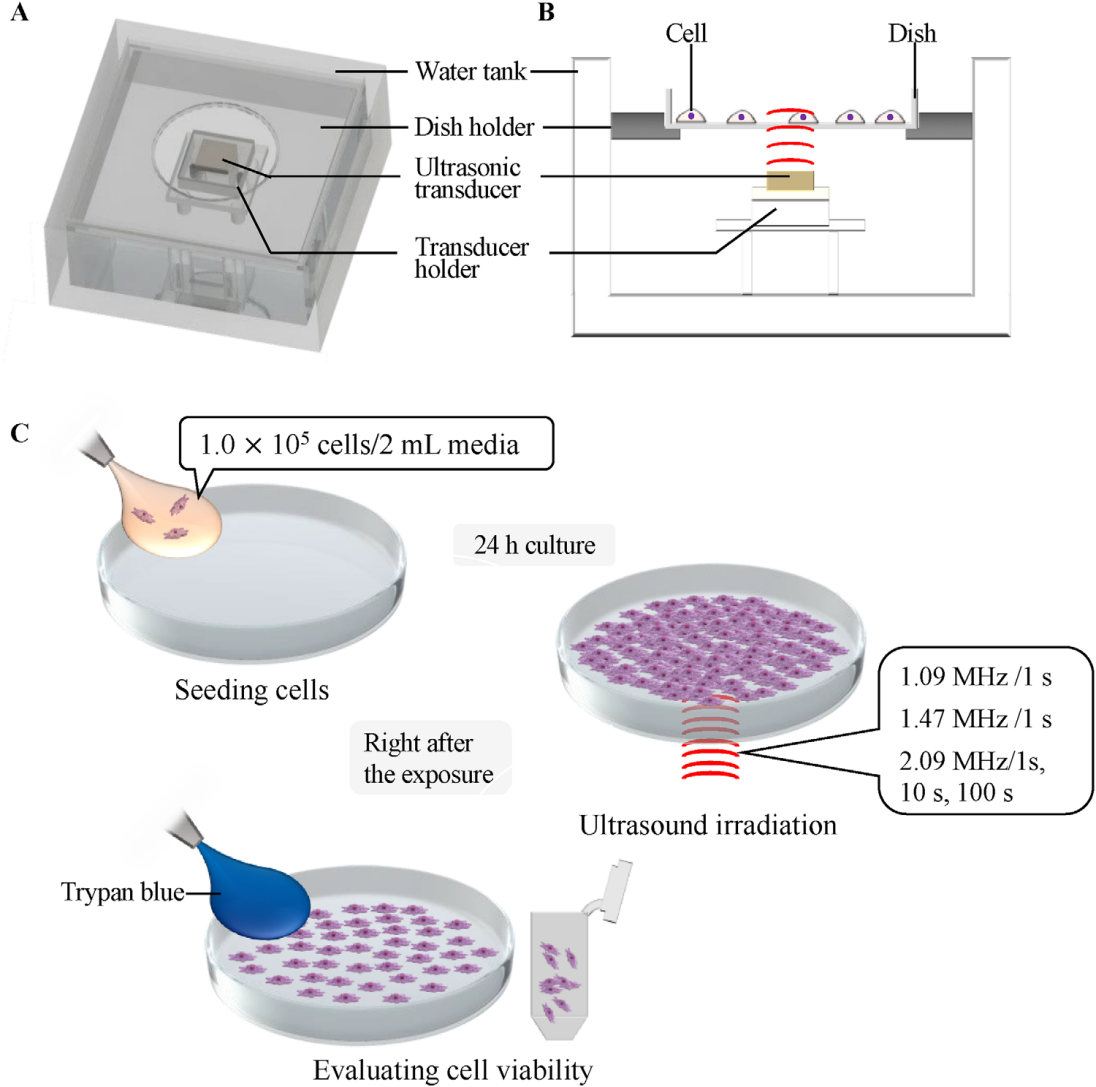
The experimental setup and procedure. The overview (A) and cross‐sectional view (B) of the experimental setup and the experimental procedure (C) are shown. In the experiment setup, the ultrasound transducer is replaceable to investigate the effect of vibration frequencies on cell viability.

Figure 1A,B provides an overview and cross‐sectional view of the developed ultrasound irradiation system. The system consists of a ø35 dish, a dish holder, an ultrasonic transducer, a transducer holder, and a water tank. For this system, three types of piezoelectric transducers (C213, Fuji Ceramics Corporation, Shizuoka, Japan) with different MHz‐range resonance frequencies widely used in bioengineering research were employed as ultrasonic transducers. The ultrasound generated by the ultrasonic transducer propagated to the center of the dish via water (Figure 1B). The experimental procedure using the setup is shown in Figure 1C.

### 2.2 Evaluation of the Vibration Characteristics of the System

**FIGURE 2:**
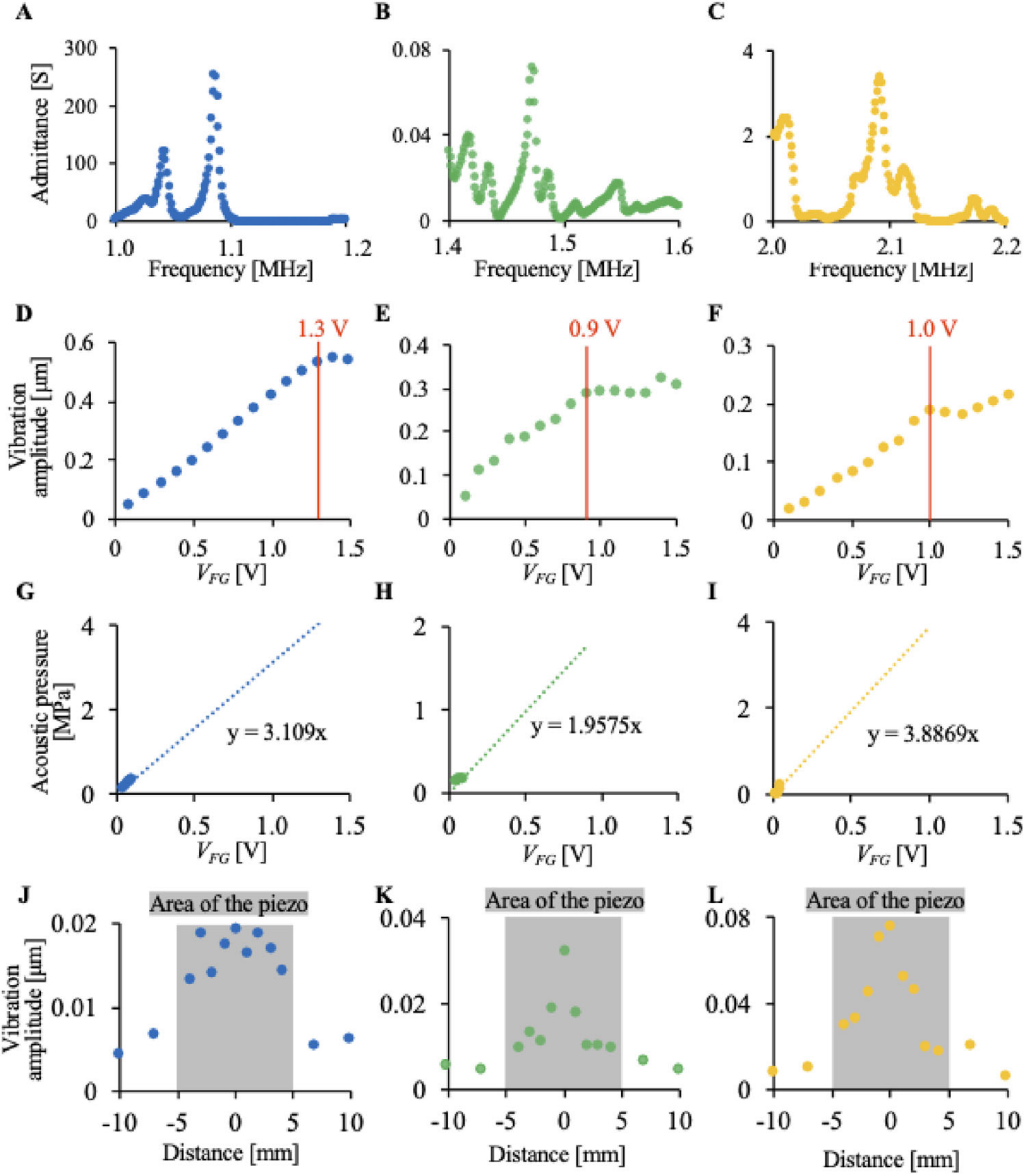
The driving condition of the setup with three different ultrasound transducers having the same irradiation area but different driving frequencies. In this study, each transducer was named UT_1.09_ (evaluated in A, D, G, and J), UT_1.47_ (evaluated in B, E, F, and K), and UT_2.09_ (evaluated in C, F, I, and L), respectively. A–C: The resonance frequency of each ultrasound transducer was evaluated. D–F: The maximum vibration displacements at the culture surfaces were measured without culture media. G–I: Maximum acoustic pressure at the culture surface was evaluated and predicted with 2 mL of water simulating the culture media. J–L: The vibration distribution on the culture surface was measured with LDV with an input voltage of 0.2 V.

The resonance frequency of each ultrasonic transducer in the system and vibration amplitude on the dish surface were evaluated. The resonance frequency of each ultrasonic transducer was evaluated by measuring the electrical impedance of each ultrasonic transducer in the system using an impedance analyzer (FRA5097; NF Corporation, Kanagawa, Japan), as shown in Figure 2A–C. The resonance frequencies of the ultrasonic transducer were 1.09, 1.47, and 2.09 MHz. Thus, these frequencies were used as the driving frequencies of the ultrasonic transducers.

The relationship between the transducer input voltage and the vibration amplitude at the center of the culture surface of the dish was measured using a laser Doppler vibrometer (LDV) (LV‐1800; Ono Sokki Co., Ltd., Kanagawa, Japan), with no culture medium in the dish, as shown in Figure 2D–F. The input voltage is the voltage output from the function generator *V_FG_*. These figures show that the vibration amplitude at the center of the top surface of the dish was proportional to *V_FG_* in the range from 0 to 1.3 V, to 0.9 V, and to 1.0 V at the frequencies of 1.09, 1.47, and 2.09 MHz, respectively. Thus, we concluded that *V_FG_* in these ranges was used as the driving voltage for the ultrasonic transducers.

Furthermore, to estimate the acoustic pressure, the vibration amplitude at the center of the top surface of the dish was measured using 2‐mL of water. Since the insertion of the hydrophone to the small chamber may affect the acoustic field, we predicted the acoustic pressure by LDV in non‐invasive manner. Although effects of acousto‐optic interaction on laser displacement measurements have been reported in previous studies [22], a change in the refractive index is not expected to have a significant impact on displacement measurements in our experimental setup. In this setup, the laser light path is aligned with the ultrasound propagation direction, and the expansion (positive pressure) and rarefaction (negative pressure) phases associated with ultrasound passage are expected to mutually weaken each other, resulting in only a very small net change in the refractive index. If there is such a huge change in the acoustic field, all the cells will be detached [23, 24, 25]. When an input voltage above a certain level was applied to the ultrasonic transducer, the water surface was raised, disturbing the vibration amplitude measurement with the LDV. Thus, to calculate the acoustic pressure from Figure 2 The driving condition of the setup with three different ultrasound transducers having the same irradiation area but different driving frequencies. In this study, each transducer was named UT_1.09_ (evaluated in A, D, G, and J), UT_1.47_ (evaluated in B, E, F, and K), and UT_2.09_ (evaluated in C, F, I, and L), respectively. A–C: The resonance frequency of each ultrasound transducer was evaluated. D–F: The maximum vibration displacements at the culture surfaces were measured without culture media. G–I: Maximum acoustic pressure at the culture surface was evaluated and predicted with 2 mL of water simulating the culture media. J–L: The vibration distribution on the culture surface was measured with LDV with an input voltage of 0.2 V. The vibration amplitude, *V_FG_* in the range in which the rise of the water surface did not disturb the measurement was used to measure the vibration amplitude with 2‐mL of water. Subsequently, the acoustic pressure outside the range calculated from the measured vibration amplitude was estimated by extrapolation based on the plots (Figure 2G–I). It was assumed that the vibration on the culture surface increases linearly, even with water. To calculate the acoustic pressure of ultrasound irradiated to cells, acoustic pressure, *p*, in the vertical direction relative to the surface of the ultrasonic transducer is given by:

(1) $$\begin{array}{c} p=\sqrt{2}\;\pi f\rho \mathrm{cA} \end{array}$$

where *f*, *c*, ρ, and *A* represent the frequency, the speed of sound, the density of the liquid, and the vibration amplitude, respectively [35]. From Equation (1), the acoustic pressure was calculated using the vibration amplitude of the top surface of the dish. Figure 2D–F shows the relationship between *V_FG_* and vibration amplitude. Due to the assumption of linear increase of vibration amplitude to the input voltage, approximate formulas, whose proportional constants are 3.11, 1.96, and 3.89 MPa/V for the transducers with the driving frequencies of 1.09, 1.47, and 2.09 MHz, respectively, were given, since we did not change the driving frequency and the acoustic field affecting the resonance frequency during this study. The ultrasound transducers were named UT_1.09_, UT_1.47_, and UT_2.09_.

The distribution of the vibration amplitudes at the top surface of the dish was also measured in Figure 2J–L. The width of the square ultrasonic transducer was 10 mm. This figure shows that the ultrasound propagated into the dish through the bottom of the dish.

### 2.3 Temperature Increases Owing to the Ultrasound Exposure

**FIGURE 3:**
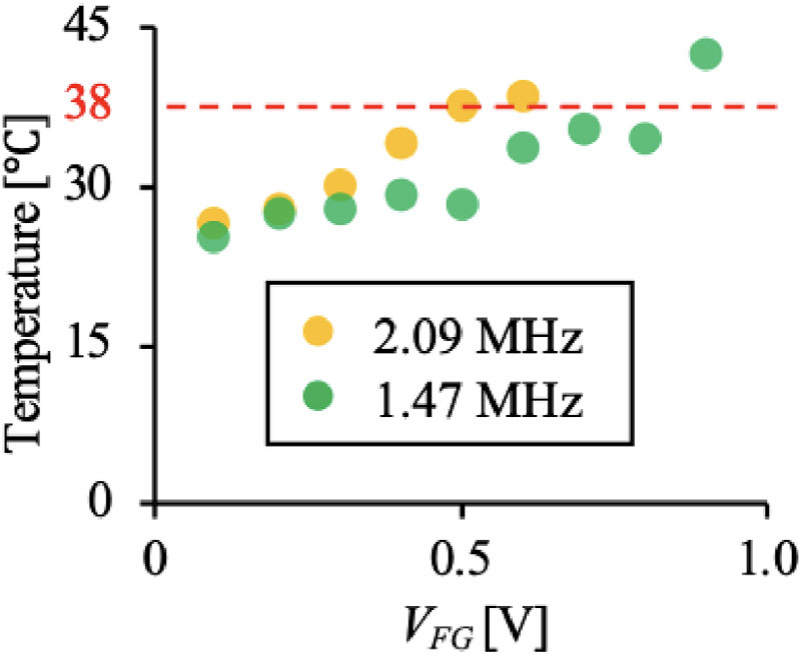
The temperature variations were measured with certain conditions. Since media temperature rises over 38°C with 100 s of ultrasound exposure using UT_1.47_ and UT_2.09_, we evaluated the temperature variation with 100 s ultrasound exposure.

The temperature of 2 mL of the culture medium subjected to ultrasonic exposure in the dish was measured using a thermographic camera (CPA‐E6; CHINO CORPORATION, Itabashi, Tokyo, Japan). *V_FG_* of 1.3 V, 0.9 V, and 1.0 V were input into UT_1.09_, UT_1.47_, and UT_2.09_ with the ultrasonic irradiation time of 1, 10, or 100 s. The temperatures measured after ultrasound irradiation are listed in Table 1. This table shows several conditions in which the temperature was increased beyond 38°C. Note that the temperature after a 100‐s ultrasound irradiation at 2.09 MHz could not be measured because the dish melted due to ultrasound‐induced heat generation. Notably, the melting point of polystyrene, which is the material of a culture dish, is around 100°C. Since cells are damaged by heat at a temperature beyond 38°C [26], these increasing culture temperature conditions were not used in the experiment, which let us focus on the ultrasound‐induced mechanical damage on cells. Thus, the relationship between *V_FG_* and the temperature after 100‐s irradiation of ultrasound at 1.47 or 2.09 MHz was investigated, as shown in Figure 3, which demonstrated that the temperature did not exceed 38°C with *V_FG_* of 0.8 and 0.7 V with UT_1.47_ and UT_2.09_. Therefore, *V_FG_* not exceeding 0.8 and 0.5 V were used along with the conditions of irradiating 1.47‐MHz ultrasound for 100 s and 2.09‐MHz ultrasound for 100 s.

**TABLE 1:** Temperature variation with each exposure time with each ultrasound transducer.

	1 s	10 s	100 s
2.09 MHz	31.3	33.9	Dish melted
1.47 MHz	31.8	32.7	42.5
1.09 MHz	33.4	33	35.4

*Note:* The input voltages of 1.3, 0.9, and 1.0 V are applied to UT_1.09_, UT_1.47_, and UT_2.09_, respectively.

### 2.4 Ultrasound‐Induced Damage on In‐Vitro Cultured Cells

**FIGURE 4:**
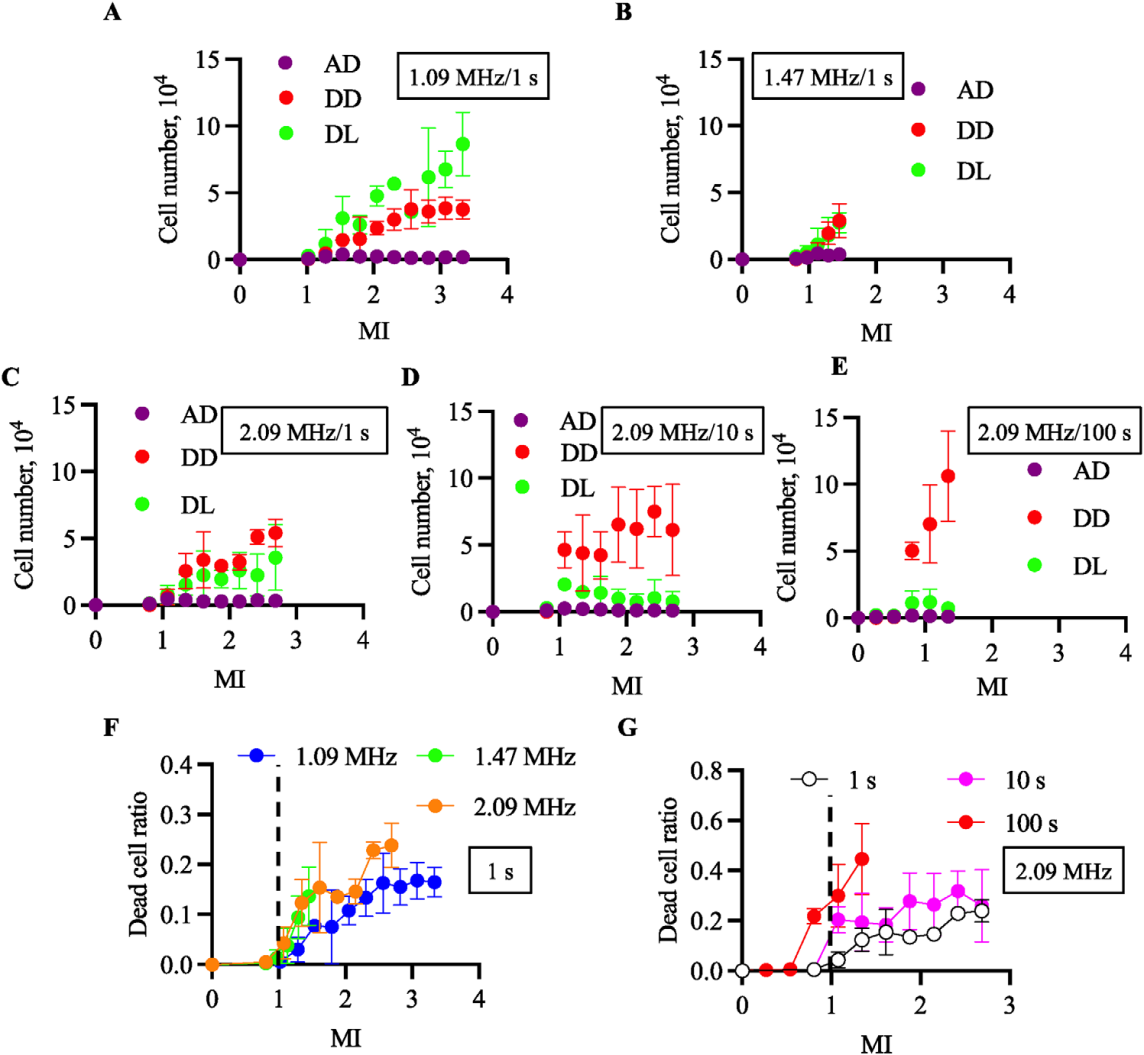
Cell viability was evaluated with ultrasound exposure to the ultrasound with each condition. The relationships between the MI and dead cell ratio were measured. The conditions and the numbers of live and dead cells are shown in A–E (*N* = 3, mean ± SD), and the ratio of dead cells was integrated into F and G (*N* = 3, mean ± SD). With an exposure time of 1 s, UT_1.09_ (A), UT_1.47_ (B), and UT_2.09_ (C) were used. Using UT_2.09_ with exposure times of 1 s (C), 10 s (D), and 100 s (E). The data were integrated with the exposure time of 1s (F) and a driving frequency of 2.09 MHz (G) to show the effect of driving frequencies and exposure time on cell viability. AD indicates adhered dead cells; DD, detached dead cells; DL: detached Live cells.

The cells were irradiated with ultrasound at different frequencies, acoustic pressures, and durations to evaluate ultrasound‐induced damage for in vitro‐cultured cells. The experimental conditions and procedure are shown in Figure 1C. Chinese hamster ovary (CHO) cells, commonly used in pharmaceutical research and industry, have been employed as a model for cultured cells [27]. For the experiment, 1.0 × 10^5^ cells were seeded with 2 mL of culture medium into a cell culture dish with a diameter of 35 mm and cultured for 1 d in a humidified 5% CO_2_ incubator at 37°C. After incubation, the culture medium was removed, and 2 mL of fresh culture medium was added to the dish. The dish was then placed on an ultrasonic irradiation system, exposing the cells to ultrasound. *V_FG_* was varied from 0.4 to 1.3 V at the frequency of 1.09 MHz, from 0.5 to 0.9 at 1.47 MHz, or from 0.3 to 1.0 V at 2.09 MHz, and the duration of ultrasonic irradiation was 1 s (Figure 4A–C). On the other hand, three exposure times were applied with the frequency of 2.09 MHz (Figure 4C–F). The ultrasound output is shown as the MI in each figure. Figure 4A–E shows the number of cells under each condition: detached and dead, detached and live, and adhered and dead cells. It has been shown that most dead cells are suspended in a culture media. The number of detached cells, either live or dead, increased with the MI at certain driving frequencies and durations. Furthermore, the ratio of dead cells to the total detached cells tended to increase with driving frequency or exposure time, while the other was fixed. The results showing the dead cell ratio to all cells are summarized in Figure 4F,G.

Simply integrating the number of dead cells regardless of the adhesion condition with a certain driving frequency, the number of detached cells increased along with increased MI and exposure time.

**FIGURE 5:**
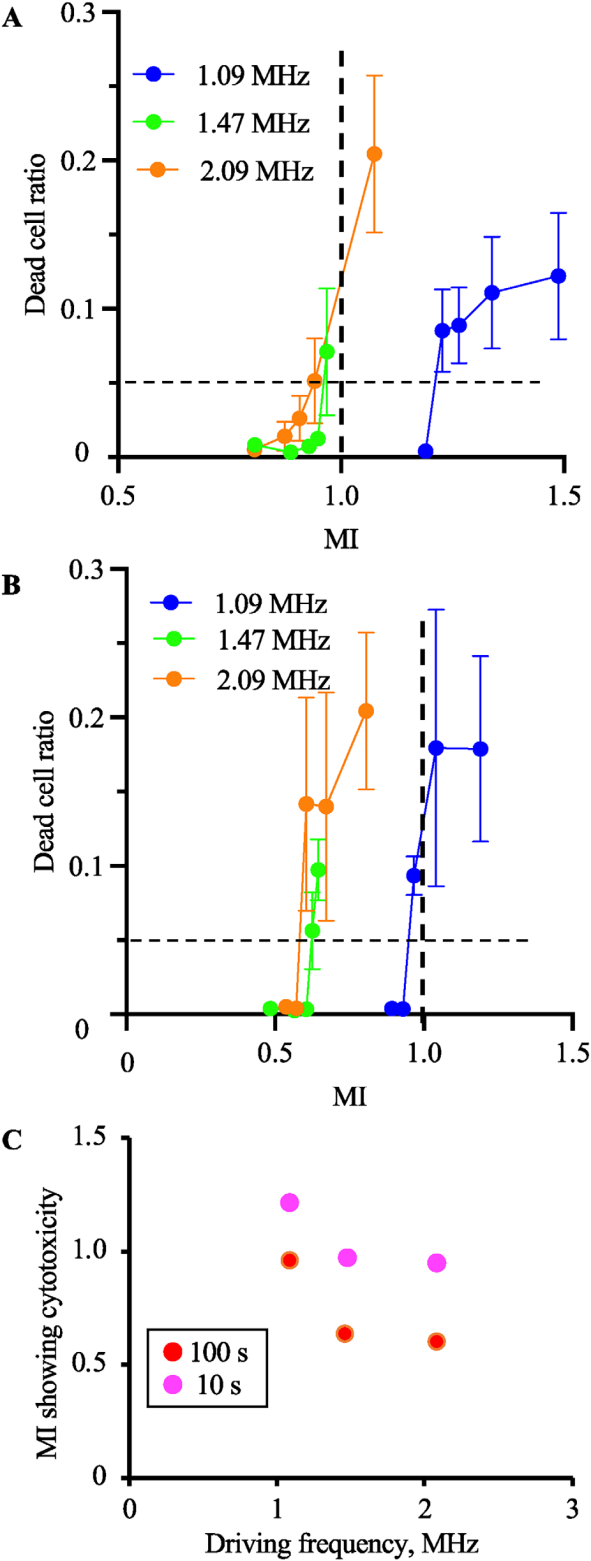
Cell viability was evaluated with 10 s (A) and 100 s (B) of ultrasound exposure (*N* = 3, mean ± SD). The relationships between dead cell ratio and MI were measured with a narrower range of MI than in Figure 4 to define the threshold. UT_1.09_, UT_1.47_, and UT_2.09_ were used. The threshold of the MI starting to show cytotoxicity with each condition was shown (C).

As an indicator of cytotoxicity, we applied a 5% dead cell ratio in this study, and the threshold of cytocompatible ultrasound conditions was evaluated by ultrasound exposure. Figure 5A,B shows the ratios of dead cells after ultrasound exposure for 10 and 100 s, respectively. Figure 5C shows the cytotoxicity of MI under each treatment condition. To obtain this figure, we calculated the point of intersection of the figure line and the *y* value of 0.05 in Figure 5A,B. The MI threshold showing cytotoxicity decreased with increasing ultrasound frequency and exposure time. This result indicates that the cytocompatible ultrasound condition in vitro is not defined only by MI and that ultrasound frequencies and exposure times are important factors.

### 2.5 Cell Distribution on the Dish After Ultrasonic Irradiation

**FIGURE 6:**
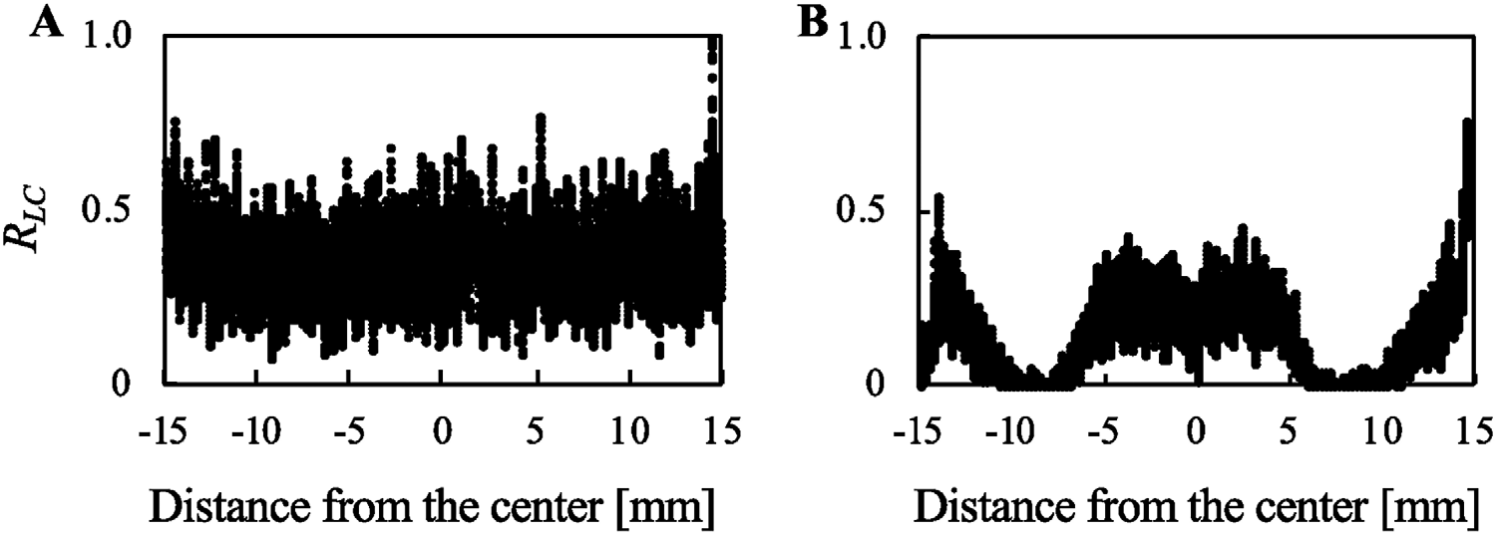
The distribution of live cells adhering to culture surfaces without (A) and with (B) ultrasound exposure of 1 s using UT_2.09_. Ultrasound with an MI of 2.7 was applied. *R*_LC_: Ratio of live cell area to the maximum live cell area in the image of A.

The distribution of live cells after 1‐s of ultrasonic irradiation with an input voltage of 1.0 V to UT_2.09_ was compared with the distribution without ultrasound exposure, as shown in Figure 6. The live cell distribution shown in Figure 6B did not correspond to the vibration distribution shown in Figure 2J, indicating that the vibration amplitude did not simply show the cytotoxicity.

## 3 Discussion

In this study, we investigated the effects of MI in cell cultures. An ultrasound irradiation system for cultured cells was developed to demonstrate the validity of using MI in vitro. Although the induced cell damage under each ultrasound exposure condition was evaluated using CHO, this device has no limitations in culturing cell species. As shown in Figures 4 and 5, the higher the driving frequency or longer the driving time, the more injured the cells. Furthermore, when focusing on an MI of 1 as a threshold for safe driving conditions [28], more than 20% of cells were dead with driving durations of 10 and 100 s (Figure 4G). Although there is no clear threshold for the definition of cytotoxicity in the literature, the MI values reaching the tentative threshold of 5% in this study decreased with increasing driving frequency and ultrasound exposure time. Based on these results, we concluded that the cytotoxicity of in vitro ultrasound irradiation is determined by multiple factors, including MI, exposure time, and driving frequency. Cytotoxicity will result from nonlinear interaction between different phenomena: acoustic, hydrodynamic, thermodynamic, and biological. Here, we show from the present experiments that cytotoxicity cannot be analyzed by a single empirical parameter of MI and can depend on multiple dimensional parameters. Since the evaluation of cytotoxicity with ultrasound may be avoided in vivo due to ethical concerns, the developed device can give good references.

MI is not the only index to be considered in vitro, as mentioned above; hence, the factors causing cell damage in our study are discussed below. Cell death can be classified as necrosis or apoptosis. Although mechanical vibration has been known to trigger apoptosis even with mild stimulation [17, 29], in our study, cells should be killed by necrosis. This is because apoptosis generally requires a long time after stress [30]. Potential factors that cause cell death include temperature increments, cavitation, deformation, acoustic radiation pressure, and acoustic streaming. As shown in Figure 3, no temperature increase was observed in our experiment. The acoustic pressure and deformation are approximately 4 MPa and less than 0.5 µm as shown in Figure 2, respectively, which should not solely cause necrosis from the previous research [31, 32, 33]. On the other hand, cavitation should be one of the factors, and previous studies have reported that MHz‐frequency ultrasound can cause cavitation [34, 35]. An MI beyond 1.0 may cause cavitation [36]. Supplementary Note 1 in the supporting information shows the presence of cavitation. Furthermore, the threshold, which generates cavitation, can be lowered owing to heterogeneous cavitation nuclei [37]. In this study, because proteins and air in culture media, cells, or cell debris can be nuclei, cavitation might have occurred in our experimental setup, even with an MI of less than 1.0. The presence of nanobubbles or microbubbles in water has been reported previously [38, 39]. Previous work has also reported that the ultrasound even with 1 MHz or less frequency nanobubbles or microbubbles could be generated [40]. Further, even with the same MI, the threshold of cavitation occurrence can be lower by the exposure time due to rectified mass diffusion and Bjerknes forces [41, 42, 43, 44, 45]. Although cavitation may not occur with a short exposure time owing to the small diameter of the nanobubbles in the media compared to the driving frequency of the ultrasound, a longer exposure time allows bubbles to grow and cause cavitation [46, 47]. However, although the threshold of cavitation occurrence should increase with an increase in the ultrasound frequency [36], a higher frequency seems harmful to the cells in our study. This indicates that there is a factor other than cavitation that induces cell death. Acoustic streaming, which could not be evaluated in this experimental setup owing to its design but should exist in our setup, is another factor causing cell death. The existence of acoustic streaming, especially Eckart streaming, could be predicted because all ultrasound wavelengths used were shorter than the thickness of the culture media (2.2 mm). Note that even for 1.09 MHz ultrasound, which has the longest wavelength, the wavelength in water is approximately 1.4 mm. Acoustic streaming becomes more intense with increased driving frequency, as reported in the literature [48, 49, 50]. The increased absorption rate of ultrasound along with the frequency increase also enhances the generation of acoustic streaming [51, 52]. If the driving frequency increases with the same MI, acoustic pressure increases, which also enhances acoustic streaming. Furthermore, because bubbles absorb ultrasound, which results in enhanced acoustic streaming, a longer exposure time may lead to enhanced acoustic streaming [53]. The strong shear stress caused by the media flow injures the cultured cells [54]. Hence, the potential factors that may injure cells in our experimental setup were predicted to be cavitation and acoustic streaming.

Although cavitation and acoustic streaming are scientific factors that injure cells, many of them affect cell durability in vitro. Variations in the culture environment, such as monoculture, 2D culture, culture media, and relatively lower cell density compared to in vivo conditions, could be factors. Furthermore, cell detachment is a trigger that should be considered when ultrasound is applied to cultured cells. In vitro cells can be detached by ultrasound exposure, and acoustic streaming is one of the factors that detach cells [24, 55, 56]. Once cells are detached, they experience a unique mechanical stress compared to in vivo conditions, which may harm the cultured cells. Cells floating in a spinner flask are known to be damaged by strong agitation [57], which indicates that floating and agitated cells could be injured in this study. In particular, in a previous study that reported suspension cultures with ultrasound exposure, cells were injured by a certain intensity of ultrasound [37]. The cell distribution is shown in Figure 6B; the live and adhered cells in the area with the largest vibration amplitude on the culture surface (the center of the culture surface) showed better viability than those in the area on the culture surface with smaller vibration amplitude. Compared to the center of the culture surface, other areas should exhibit stronger streaming, which may injure cells. This prediction on the entire streaming is supported by previous studies that show the upward streaming underneath the acoustic fountain [20, 58]. Above all, although cavitation and acoustic streaming should be direct triggers to induce cell death, every culture condition and cell function affects cell durability. Hence, using this setup, an experimental approach was adopted to investigate the ultrasound conditions that induce cell death.

Our device employed general culture dishes, which allowed us to investigate the cytotoxicity of ultrasound in typical cell culture situations. The current setup has a distribution of acoustic phenomena and cell density, which provide a reality for in vitro culture. However, by realizing homogeneous experimental conditions in the future, cell durability or response to ultrasound exposure can be strictly investigated from a scientific viewpoint. We can employ the reported techniques to achieve a homogeneous culture density, making cell conditions homogeneous [59, 60, 61]. Further, experimental setups in which the cultured cells can be exposed to uniform ultrasound stimulation or the distribution of the ultrasound stimulation can be governed should be developed in future studies. As mentioned above, this study shows a fundamental idea to investigate the cytotoxicity of ultrasound in vitro and develop sonochemistry in the bioengineering research field; a novel device where cell responses to ultrasound can be investigated should be developed in the future.

## 4 Methods

### 4.1 Evaluation of the Vibration Characteristics of the System

The water tank was filled with water, and a dish containing 2 mL of water was placed on the dish holder to replicate the conditions of the experiment using cells to measure the resonance frequency. The relationship between the AC input frequency and the admittance of the ultrasound transducer was measured at an input voltage of 1 V_pp_.

### 4.2 Cell Preparation

A CHO cell line (CHO‐K1, RCB0403; Riken BioResource Center, Ibaraki, Japan) was used for all experiments. The CHO cells were cultured in Ham's F‐12 medium (a‐ MEM Ham's F‐12; Wako, Tokyo, Japan) supplied with 10% fetal bovine serum (S1820; Biowest SAS, Nuaille, France) in a 5% CO_2_ humidified‐atmosphere incubator at 37°C. Cell passaging was performed by applying 0.05% trypsin‐EDTA (25300, Life Technologies, CA, USA), followed by pipetting.

### 4.3 The Evaluation of Cell Viability

After ultrasonic irradiation, the culture medium in the dish was collected, and the number of live and dead cells in the supernatant and the number of dead cells on the culture surface were measured using trypan blue solution (0.4%) (Thermo Fisher Scientific) on a hemocytometer. Then, 250 µL of trypan blue solution was added to the dish to dye the dead cells adhered to the dish. The number of dead adherent cells was counted as follows: An image of the entire dish was taken, and the number of adhered dead cells stained with trypan blue was counted using the MATLAB image processing toolbox (Image Processing Toolbox; MathWorks, Natick, MA, USA).

### 4.4 The Evaluation of Cell Distribution

After removing the medium, the cells were washed three times with 1 mL PBS. After removing PBS, the cytoplasm or nuclei of the cells were stained with calcein‐AM (C0875; Sigma‐Aldrich, St. Louis, MO, USA) for 30 min and washed again with PBS. Fluorescence images of the cells were captured using an inverted microscope. The size of the captured image was 1.63 × 29.8 mm^2^ and the center of the image was aligned with the center of the dish. The captured fluorescent images were analyzed using Image J as follows: the captured images were binarized, which turned the stained cytoplasm or nuclei of the cells black. The column averages of the luminance values at points along the *x*‐axis were then obtained. Note that the column average is the average of the luminance values of all the pixels vertically distributed at a point on the *x*‐axis.

## Author Contributions

Conceptualization: T.O., C.I., and K.T.; methodology: T.O. and K.T.; software: K.A.; validation: Y.K. and K.T.; formal analysis: T.O. and K.A.; investigation: T.O. and C.I.; resources: K.T.; data curation: C.I. and Y.K.; writing–original draft preparation: T.O. and C.I.; writing–review and editing: Y.K., K.A., and K.T.; visualization: C.I.; supervision: K.T.; project administration: K.T.; funding acquisition: C.I. and K.T. All authors have read and agreed to the published version of the manuscript.

## Conflicts of Interest

The authors declare no conflicts of interest.

## Supporting information



Supporting Information

## Data Availability

The data that support the findings of this study are available from the corresponding author (K.T.) upon reasonable request.
