# Supplementary material for: Influence of Exposure Time and Driving Frequency on Cytotoxicity in In Vitro Ultrasound With Constant Mechanical Indices
Source: Eng Life Sci. 2025 May 15;25(5):e70011. doi: 10.1002/elsc.70011 (PMC12079088; doi:10.1002/elsc.70011)
Supplement: Supplementary file 1 — Supporting Information [file ELSC-25-e70011-s001.pdf]

Influence of exposure time and driving frequency on cytotoxicity in in vitro ultrasound with constant mechanical indices

Taigo Oyama[1†], Chikahiro Imashiro[2,3†], Yuta Kurashina[4], Keita Ando[2], Kenjiro Takemura[2*]

[1]School of Science for Open and Environmental Systems, Graduate School of Science and Technology, Keio University, Yokohama, Japan. [2]Department of Mechanical Engineering, Keio University, Yokohama, Japan. [3]School of Engineering, The University of Tokyo, Tokyo, Japan. [4]Division of Advanced Mechanical Systems Engineering, Institute of Engineering, Tokyo University of Agriculture and Technology, Tokyo, Japan.

†These authors contributed equally to this work.

* Corresponding author: K.T.: takemura@mech.keio.ac.jp

Keywords: Cavitation, Ultrasound, Sonochemistry, Cell culture.

Supplementary Note 1

To evaluate the occurrence of acoustic cavitation, the amount of dissolved oxygen (DO) in the culture medium after 100 s of ultrasonic irradiation was measured. Notably, the frequency and acoustic pressure of the ultrasound were 2.09 MHz and 1.94 MPa, respectively. The amount of DO in the culture medium after 100-s of ultrasonic irradiation was significantly lower than that not exposed to ultrasound (Fig. S1). A previous study demonstrated that the amount of DO decreased because of rectified diffusion when acoustic cavitation occurred. Thus, the results demonstrated that acoustic cavitation occurred in the dish during ultrasonic irradiation.

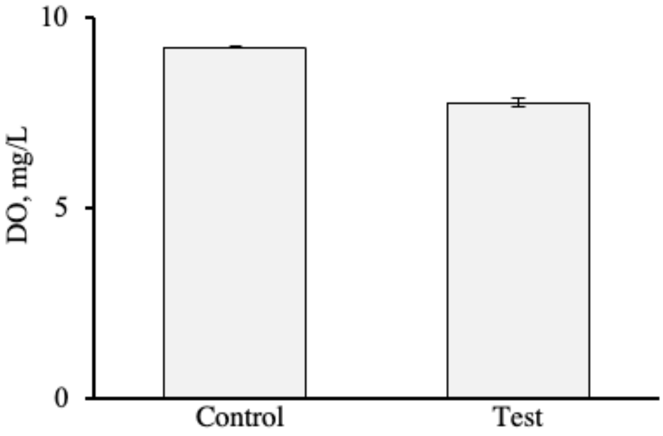

Figure S1 The amount of dissolved oxygen (DO) in the media after 100 s of ultrasound irradiation. The frequency and the acoustic pressure of the ultrasound were 2.09 MHz and 1.94 MPa, respectively. Control and Test indicate the samples with and without ultrasound irradiation, respectively.
